# Uncovering the Pharmacological Mechanism of Astragalus Salvia Compound on Pregnancy-Induced Hypertension Syndrome by a Network Pharmacology Approach

**DOI:** 10.1038/s41598-017-17139-x

**Published:** 2017-12-04

**Authors:** Liuting Zeng, Kailin Yang, Jinwen Ge

**Affiliations:** Hunan University of Chinese Medicine, Changsha, 410208 Hunan Province China

## Abstract

To uncover the pharmacological mechanism of Astragalus Salvia compound (ASC) on pregnancy-induced hypertension syndrome (PIH), to provide useful information for clinical, as well as to connect the basic and clinical by a network pharmacological approach, we used network pharmacological approach. We collected ASC’s compounds by traditional Chinese Medicine databases, and input them into PharmMapper to got their targets. Then we acquired PIH targets from Genecards and OMIM, collected the interactions of all the targets and other human proteins via String and INACT. We also constructed the network by Cytoscape and analyze it by MCODE so as to get clusters. Finally, we put all the targets of clusters into DAVID to do GO enrichment analysis. After these, four networks are constructed by Cytoscape; they are PIH network, compound-compound target network of ASC, ASC-PIH network, and compound target-PIH target-other human proteins’ PPI network. According to the results, we think that ASC may directly regulate several biological processes and their genes in “endothelial cell activation and injury” and “placental or trophoblast cell ischemia” models to treat PIH. And it may indirectly act on the rest of the biological process to treat PIH or may not.

## Introduction

Pregnancy-induced hypertension syndrome (PIH) is defined as the development of new arterial hypertension and proteinuria in a pregnant woman after 20 weeks gestation^1^. PIH occurs in 2~10% of pregnant women, which depends on its definitions and demographic studies^2–4^. The causes of perinatal woman’s death are mainly the complications of hypertension in pregnant women such as cerebrovascular accident, cerebral edema, liver rupture, renal failure and heart failure; the main pathologic mechanism of which is the endothelial cell dysfunction or multiple cytokine stimulation, including vasospasm and increased vascular reactivity, which result in the increased risks of intrauterine fetal growth retardation, stillborn fetus and premature birth^5–7^. Currently, the major pharmacologic options for PIH include aspirin, antispasmodic drugs (magnesium sulfate) and antioxidants and anti-hypertensive drugs^8^. However, their clinical use had been limited because of the uncertainty of regulation of blood pressure and the dysfunction of blood coagulation during childbirth caused by them^9^.

Therefore, many patients tend to seek complementary and alternative medicine (CAM). In China and Asian countries, herbal medicine or herb formulae, a type of CAM, had been widely used for a long time. Herbal medicine is widely used in clinical; and it is characterized by fewer side effects, long treatment cycles and slow effects^10–12^. Latest research shows that herbal medicine is able to improve fetal blood flow, intrauterine fetal growth retardation and placental microcirculation^13–15^.

Our previous study preliminary confirmed that Astragalus Salvia Compound (ASC) has a good preventive and therapeutic effect on PIH. In these studies, we find that ASC can prevent the development of PIH by preventing endothelial cell injury, promoting placental trophoblast cells and vascular endothelial cells synthesizing and secreting NO, regulating the levels of prostaglandins (PG) and endothelin (ET), and affecting placental formation and implantation, trophoblast cell apoptosis and maternal-fetal interface immunity^16–18^. However, its pharmacological mechanism has not been clarified completely.

Herbal formulae can play an integral role in the key biological process of disease development by acting on the multiple targets through its multiple components, which plays a therapeutic role^19^. However, many studies still apply the traditional research idea, “one-drug-one target-one-illness”, which ignores the multi-target and multicomponent characteristic of herbal formulae. Due to the rapid development of bioinformatics, the network pharmacology approach has become a new means to reveal herbal formulae’s molecular mechanism efficiently and systemically^20,21^. Network pharmacology studies the relationships between drugs, targets, and diseases, and shows the network of drug-targets through systematic idea. It also abstracts the interaction relationship into a network model and studies the effect of drugs on a biological network from a holistic perspective. Therefore, we use the network pharmacology method to explore the impact of ASC on PIH and its molecular mechanism from another point of view so as to provide useful information for clinical and promote connections between the basic and clinical.

## Results and Discussion

### PIH Network Analysis

#### PIH Network

**Figure 1:**
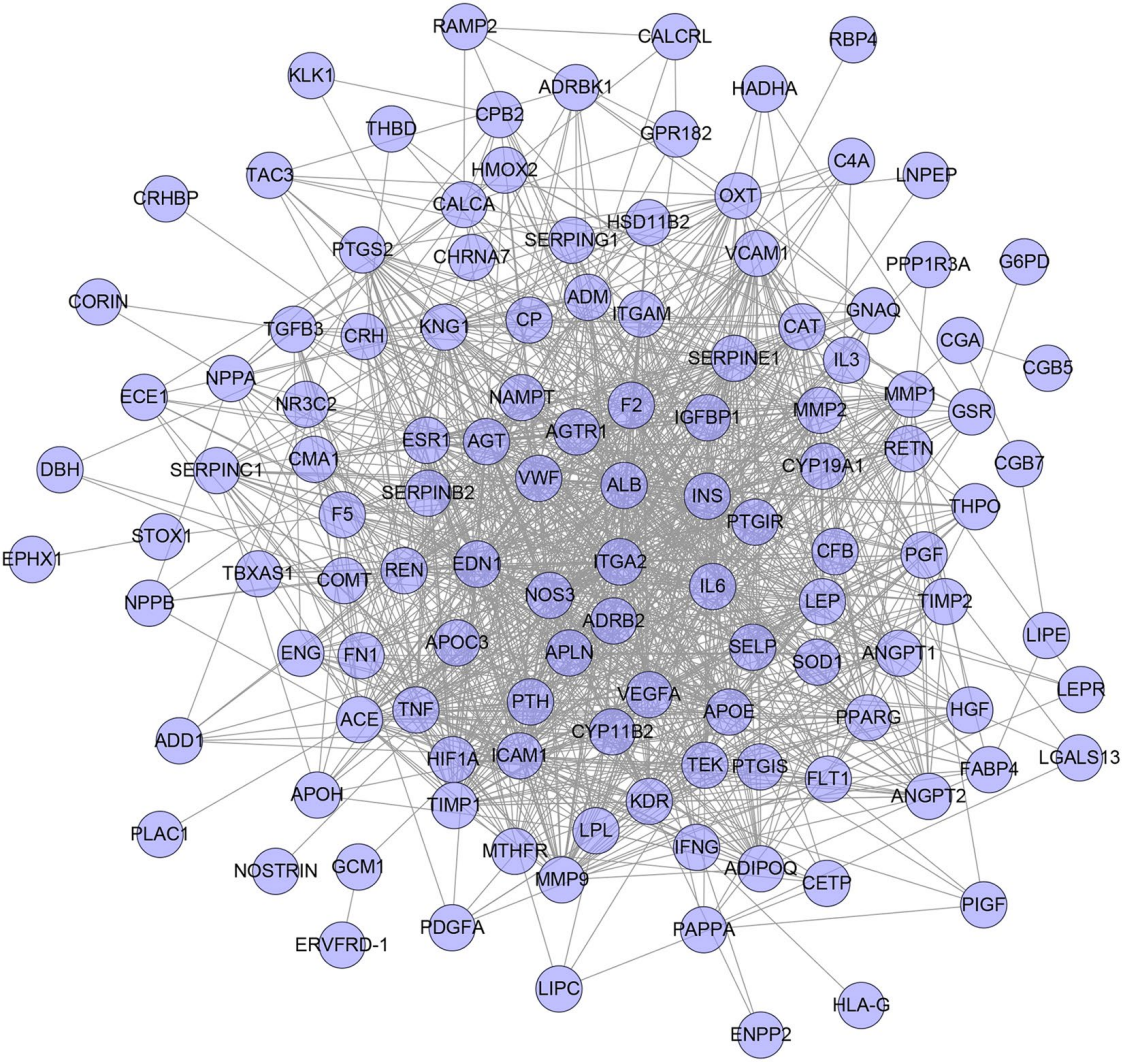
PIH targets' PPI Network.

Construct this “gene-gene interaction” network based on the data of PIH genes’ PPI and PIH genes. This network contains 113 nodes and 1142 edges (Fig. 1).

#### Clusters of PIH Network

**Figure 2:**
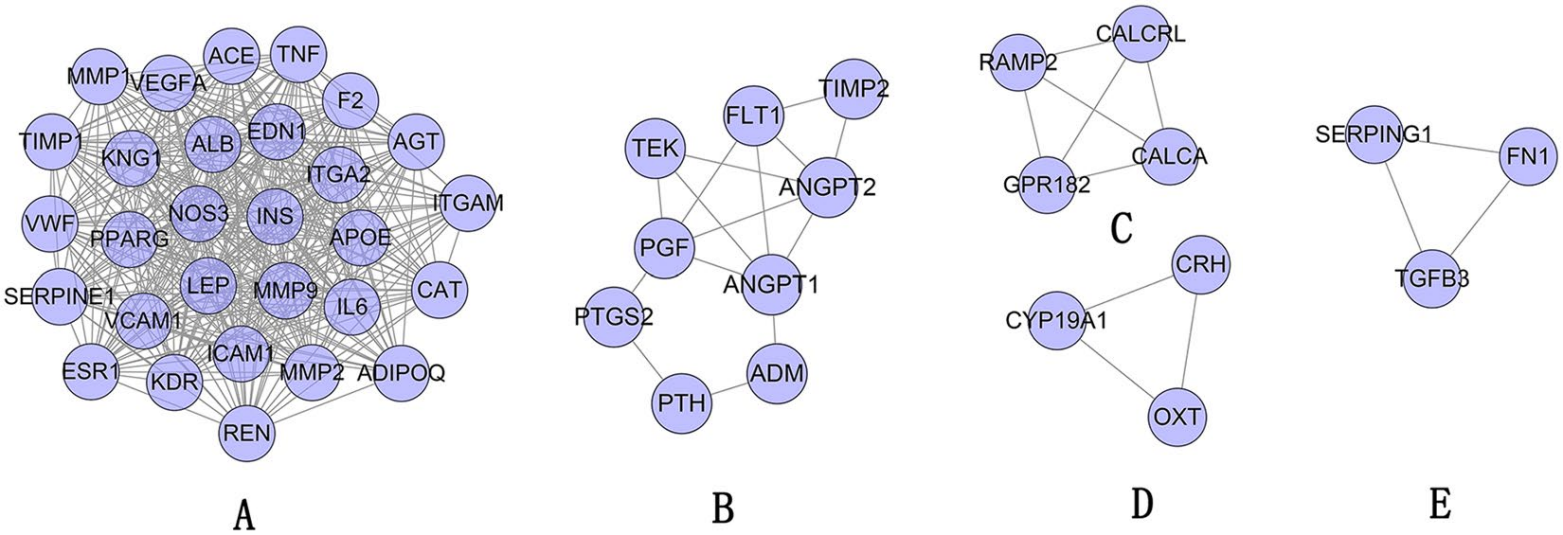
Cluster of PIH PPI network.

Analyze the network by MCODE, five clusters are returned (Fig. 2). Input these clusters into DAVID for GO enrichment analysis, several PIH-related biological processes are returned. The details are described in Table S1. And after filtering by Bonferroni <0.05, only cluster A, C, E contained significant biological processes. Take some PIH-related biological processes in Cluster A as an example:

Genes in Cluster A are related to many biological processes. According to PIH’s etiologies, these biological processes can be divided into 4 modules. (1) Endothelial cell activation and injury: (GO:0007596) blood coagulation, (GO:0030168) platelet activation, (GO:0002576) platelet degranulation, (GO:0030195) negative regulation of blood coagulation, (GO:0010544) negative regulation of platelet activation, (GO:0007597) blood coagulation, intrinsic pathway. (2) Placental or trophoblast cell ischemia: (GO:0045429) positive regulation of nitric oxide biosynthetic process, (GO:0008217) regulation of blood pressure, (GO:0043066) negative regulation of apoptotic process, (GO:0001890) placenta development, (GO:0010595) positive regulation of endothelial cell migration, (GO:0042311) vasodilation, (GO:0007263) nitric oxide mediated signal transduction, *et al*. (3) Hypoxia and oxidative stress: (GO:2000379) positive regulation of reactive oxygen species metabolic process. (4) Maternal-fetal immune tolerance disorders: (GO:0071356) cellular response to tumor necrosis factor, (GO:0001819) positive regulation of cytokine production, (GO:0045766) positive regulation of angiogenesis, (GO:1902042) negative regulation of extrinsic apoptotic signaling pathway via death domain receptors, *et al*.

Under the direction of network biology, network pharmacology and network medicine, we mine the data from public database and utilize network analysis to analyze the key genes and biological processes of PIH’s PPI network. This is to find the functional module about PIH’s pathogenesis. The results preliminarily show that PIH has a relationship with multi-gene, which increases the susceptibility. These pathogenesis-related biological processes mainly involve four functional modules: (1) Endothelial cell activation and injury; (2) Placental or trophoblast cell ischemia; (3) Hypoxia and oxidative stress; (4) Maternal-fetal immune tolerance disorders.

(1) Endothelial cell activation and injury. Research has shown that PIH patients suffer from glomerular endothelial cell hyperplasia and partial placental spiral arteries fibrinoid necrosis^22^. Its pathophysiological changes^23^ include: (1) the increased vasoconstrictor (endothelin [ET] and thromboxane A2 [TXA2]) and decreased vasodilators (NO and prostaglandin E2 [PGE2]); (2) the vascular endothelial injury caused by vasospasm aggravates; and (3) the increased secretion of procoagulant substances (TAX2 and coagulation factor [F] VIII) and the decreased secretion of anticlotting substances (PGI2, tissue-type plasminogen activator [t-PA] and antithrombin-III [AT-III]). In this research, several related biological processes were found; and after filtering by Bonferroni <0.05, the significant biological processes were gotten, such as (GO:0007596) blood coagulation, (GO:0030168) platelet activation, (GO:0072012) glomerulus vascular development. This suggests that those “significant biological processes” may be the crucial ones associated with treatment. Moreover, the gene “VEGFA” is included by many significant biological processes. And recent research has also demonstrated that angiogenic imbalance plays an important role in PIH—it shows that the most potent, pro-angiogenic protein is vascular endothelial growth factor (VEGF). VEGF is crucial for the maintenance of endothelial cell function; and reduced VEGF results in hypertension, proteinuria, and endotheliosis^24,25^. Research indicates that a VEGF antagonist soluble FMs-like tyrosine kinase-1 (sFlt-1) in the circulation of preeclamptic patients is significantly elevated^26^. This soluble form of the protein binds free VEGF, making it unavailable for signaling to its endogenous receptors. Although the exact mechanisms of sFlt-1’s upregulation are still an area being investigating, it is believed to be at least partially dependent on the activity of hypoxia inducible factor 1α (HIF-1α), suggesting changes in oxygen tension promotes angiogenic imbalance^27^.

(2) Placental or trophoblast cell ischemia. In normal placental development, spiral arteries, the high resistance vessels, are remodeled into high capacitance, low resistance vessels^28^. Early study has suggested that preeclampsia patients had inadequate remodeling of these spiral arteries^29^; the ultimate result of which is chronic ischemia in the placental tissue as the hemodynamic demand increases through gestation. The most important changes of this are the altered angiogenic balance, the activation of maternal inflammatory responses, the decreased nitric oxide bioavailbility, and the increased production of the vasoconstrictor ET-1. In this research, several significant biological processes are found, such as (GO:0001974) blood vessel remodeling, (GO:0001890) placenta development, (GO:0007263) nitric oxide mediated signal transduction.

(3) Hypoxia and oxidative stress. In normal pregnant women, lipid peroxides start significantly increasing from the second trimester to the third trimester, meanwhile placental tissue’s antioxidant ability also increases. However, PIH patients suffer from the imbalance of oxidation and antioxidant. There are mainly two explanations for mechanism: (1) Placental ischemia and hypoxia. The activity of xanthine oxidase and the content of nitrotyrosine increases in local placenta. Meanwhile, the activity of superoxide dismutase decreases^30^, while the content of lipid peroxidation products and anti-ox-LDL antibody, and the consumption of ascorbic acid increases^31^. Studies have found that the production of inflammatory cytokines, especially tumor necrosis factor α (TNF-α) and interleukin-6 (IL-6), in preeclampsia patients and preeclampsia animal models are increased^32,33^. All of these indicate that PIH patients produce excessive ROS and inflammatory cytokines. (2) Maternal factors. In PIH patients, the peroxide substrates increases and antioxidant capacity decrease. These changes are related to genetic background, environment, obesity, diabetes and diet^34^. According to this research, the biological processes “(GO: 2000379) positive regulation of reactive oxygen species metabolic process” may be the key one associated with treatment.

(4) Maternal-fetal immune tolerance disorders. PIH has a relationship with immune disorders because of that in PIH patients, spiral arteries acute atherosclerosis and fibrinoid necrosis, and perivascular lymphocytic infiltration is visible. Maternal-fetal immune tolerance disorders may be associated with these factors: (1) Alloantigen overload. (2) Abnormal expression of HLA-G and HLA-C in trophoblast cells. HLA-G and HLA-C can bind to the inhibitory receptor on nature kill (NK) cells and then induce immunosuppression, which is one of the important mechanisms of establishing maternal-fetal immune tolerance^35,36^. (3) Abnormal changes of T lymphocyte subsets. In normal pregnant women, the TH1/TH2 ratio tends to Th2. However, In PIH patients, the Th1-mediated cellular immunity strengthens; and the related trophoblast cells’ immune injury aggravates^37,38^. For instance, GO:0001819, GO:0032757, and so on.

### Compound-Compound Target Network Analysis

**Figure 3:**
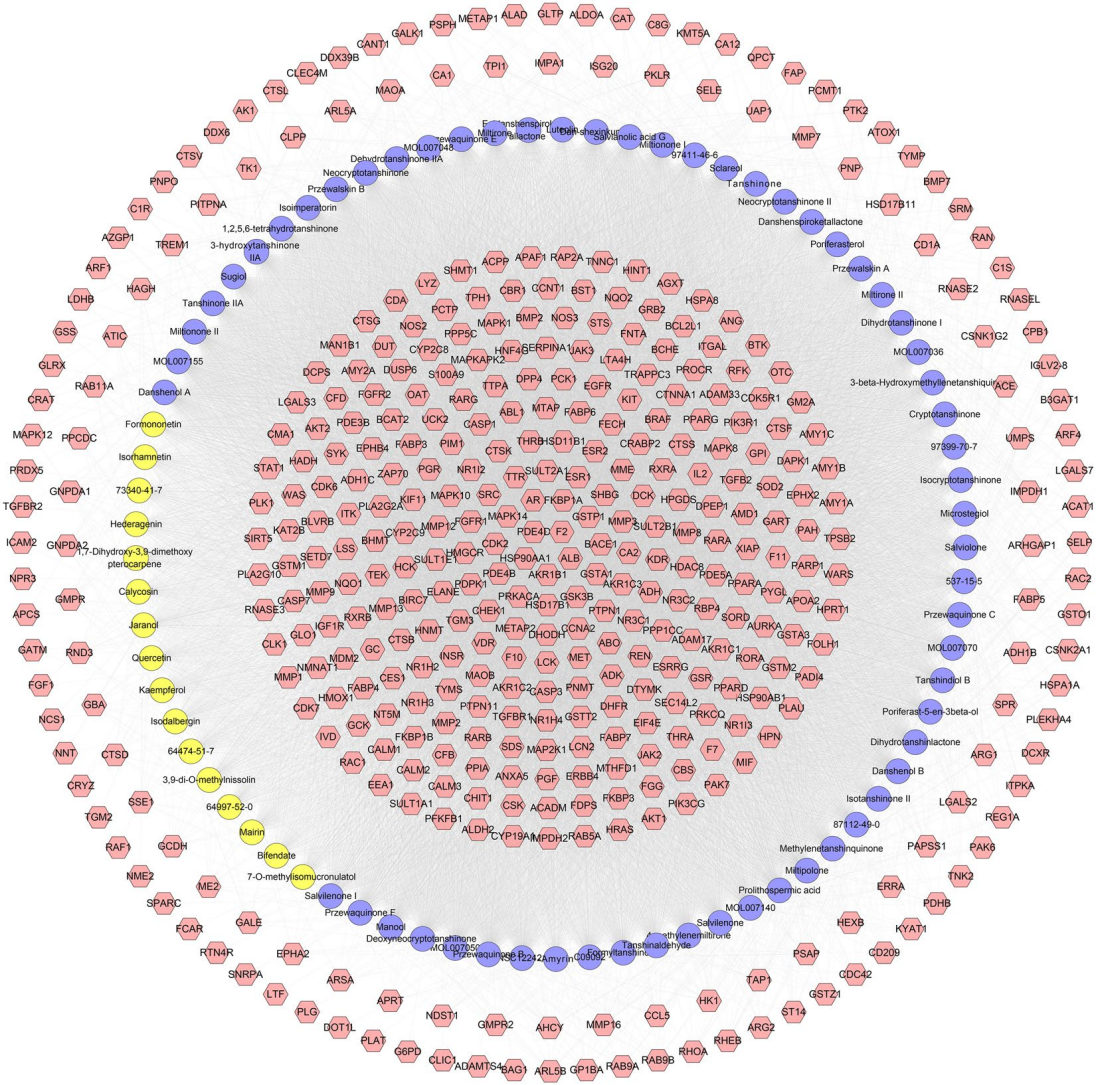
Compound-compound target network of ASC consist of 350 compound targets and 22 compounds (Pink hexagons stand for compound targets; Yellow circles and blue circles stand for compounds of *Hedysarum Multijugum Maxim.* and *Radix Salviae*, resp).

Construct network the compound-compound target network containing 509 nodes (432 compound target nodes and 77 compound nodes) and 17284 edges. It can be found in Fig. 3 that some targets are hit by many compounds, while another one can be modulated by only one compound (such as ADAMTS4, BAG1, CLIC1, DOT1L, FCAR *et al*.). For instance, AKR1B1, ALB, AR, BACE1, CDK2, F2 and so on can be controlled by all of the compounds; and both ACE and ANG can be regulated by Danshenol A, Przewalskin A, Prolithospermic acid, Salvianolic acid G, Tanshinone VI, MOL007140, Miltipolone, Przewalskin B, Isoimperatorin, Epidanshenspiroketallactone, Danshenspiroketallactone, Jaranol and 3,9-di-O-methylnissolin (Fig. 3). The details of the relationship between compounds and targets are described in Table S2.

This network shows herbal formulae’s feature of multi-compound - multi-target. The potential effect of ACS can be carried out by this network. However, we do not know whether the relationship between them is synergistic, antagonistic, or otherwise; therefore, further research is needed to clarify it.

### ASC-PIH Network Analysis

#### ASC-PIH Network

**Figure 4:**
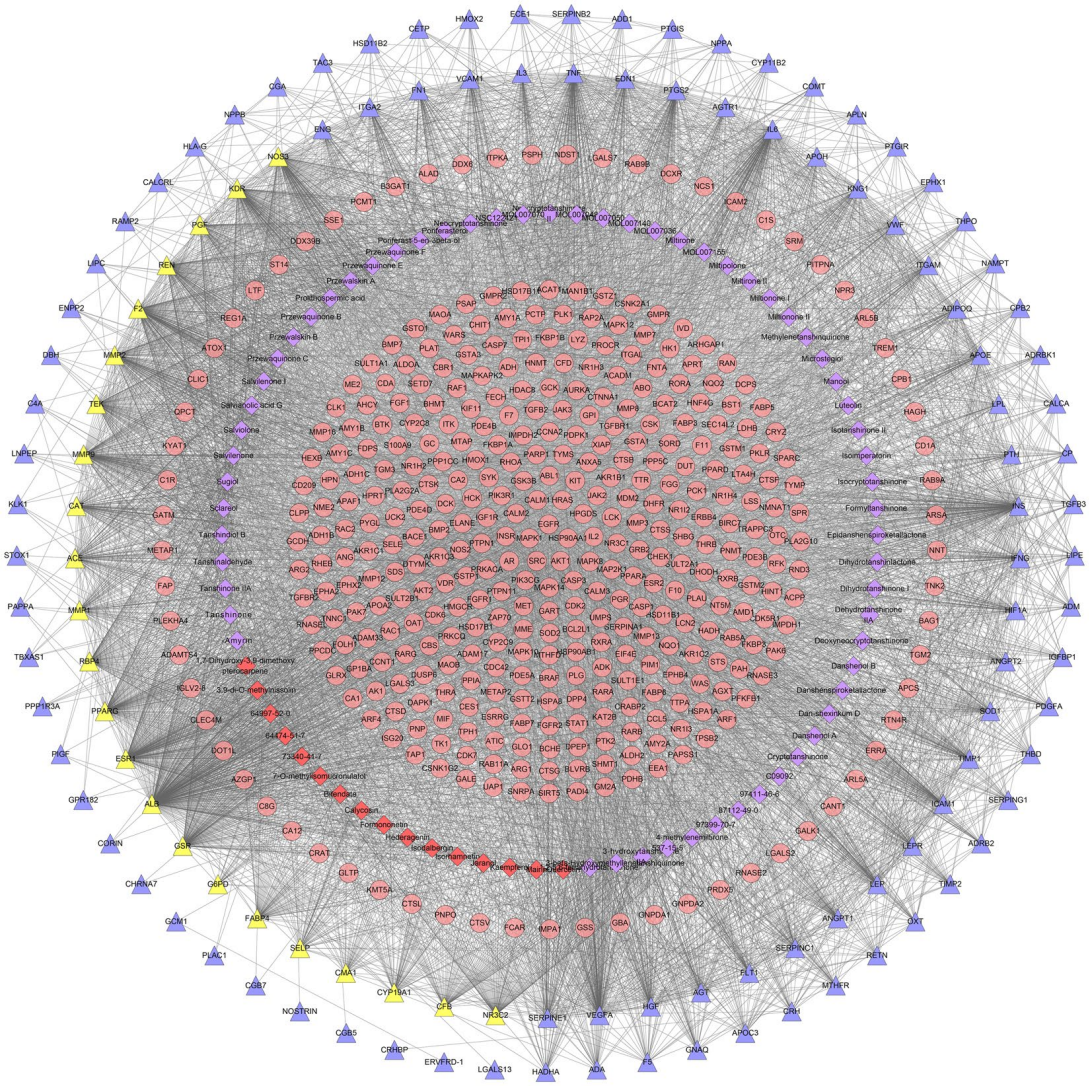
ASC-PIH network (Red diamonds and purple diamonds stand for compounds of *Hedysarum Multijugum Maxim.* and *Radix Salviae*, resp. Pink circles, blue triangles and yellow triangles stand for compound targets, PIH targets and compound-PIH targets, resp. Light lines stand for the relationship  among compounds and other nodes, dark lines stand for the relationship among PIH targets and compound-PIH targets and compound targets).

Integrate PIH network and compound-compound target network, we can get ASC-PIH network. This network contains 608 nodes and 25173 edges. Compared with PIH network, this network adds 99 nodes and 7889 edges (Fig. 4). The details of the relationship between targets, PIH targets and compound-PIH targets are described in Table S3.

#### Clusters of ASC-PIH Network

**Figure 5:**
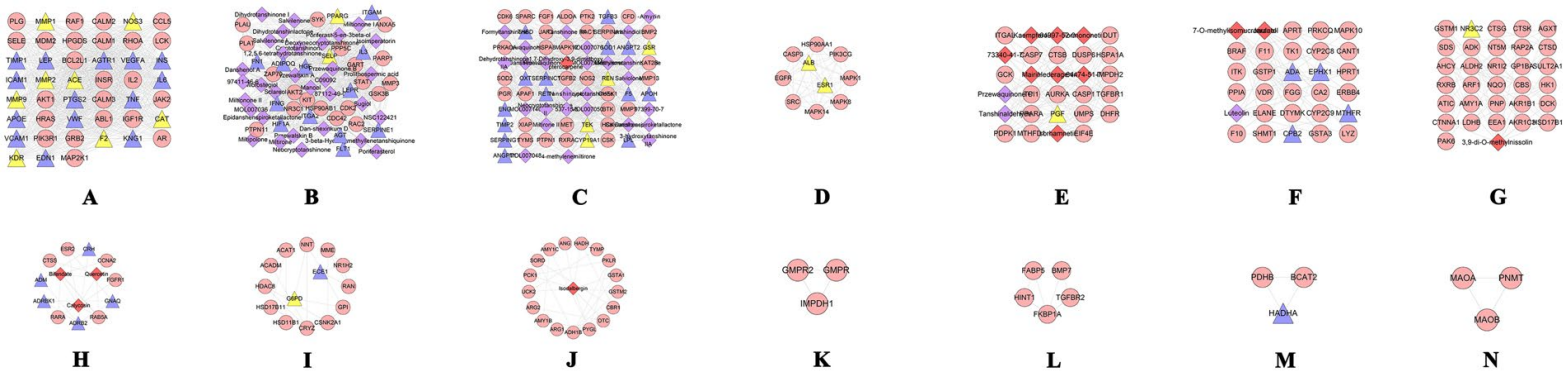
Cluster of ASC-PIH network (Red diamonds and purple diamonds stand for compounds of *Hedysarum Multijugum Maxim.* and *Radix Salviae*, resp. Pink circles, blue triangles and yellow triangles stand for compound targets, PIH targets and compound-PIH targets, resp.).

Analyze the network by MCODE, fourteen clusters are returned. It can be found that several compounds are included by clusters, such as cryptotanshinone, tanshinone IIA, kaempferol, quercetine, and so on. This suggests that these compounds play important roles in treating PIH (Fig. 5). The details of the clusters are described in Table S4.

Deal with these clusters by GO enrichment analysis. Cluster M does not return any biological processes. Cluster G, I, L, N does not return PIH-related biological processes. And after filtering by Bonferroni <0.05, only cluster A, C contained significant biological processes. Take some PIH-related biological processes in Cluster A as an example:

Genes in cluster A are related to many biological processes. According to PIH’s etiologies, these biological processes can be divided into 4 modules. (1) Endothelial cell activation and injury: (GO:0002576) platelet degranulation, (GO:0030168) platelet activation. (2) Placental or trophoblast cell ischemia: (GO:0045429) positive regulation of nitric oxide biosynthetic process, (GO:0043066) negative regulation of apoptotic process, (GO:0051000) positive regulation of nitric-oxide synthase activity, *et al*. (3) Hypoxia and oxidative stress: (GO:0071456) Cellular response to hypoxia. The details are described in Table S5.

### Compound Target-PIH Target-Other Human Proteins’ PPI Network Analysis

#### Compound Target-PIH Target-Other Human Proteins’ PPI Network

**Figure 6:**
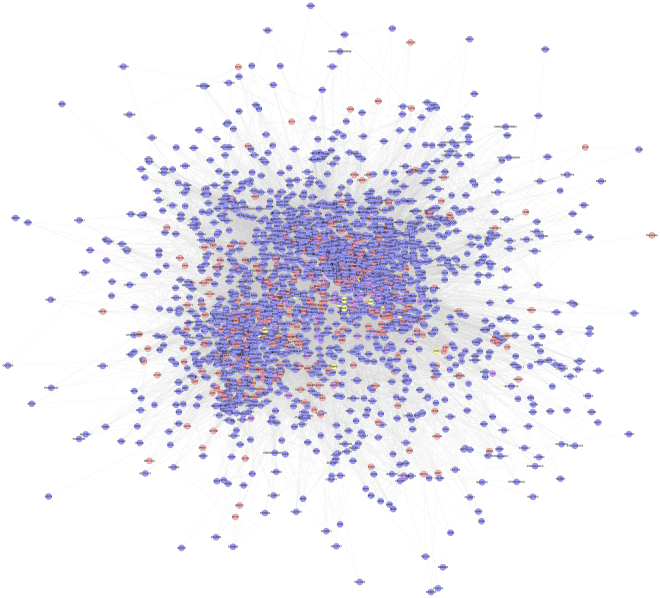
Compound target-PIH target-other human proteins’ PPI network (Blue circles, pink circles, yellow circles and purple circles stand for other human proteins, compound targets, compound-PIH targets, PIH targets, resp).

Input the compound targets into InAct and String database, several other human proteins were gotten. Compound target-PIH target-other human proteins’ PPI network is composed of 2444 nodes and 88132 edges (Fig. 6). Compared with ASC-PIH network, this network adds 1836 nodes and 62959 edges. This network is a expand network of ASC-PIH network. In these “other human proteins”, we find some PIH targets and compound-PIH targets (purple and yellow nodes), which demonstrates ACS may regulate PIH genes directly or indirectly to achieve treatment effect. The details of the relationship between compound targets and other human proteins are described in Table S6.

#### Cluster of Compound Target-PIH Target-Other Human Proteins’ PPI Network

**Figure 7:**
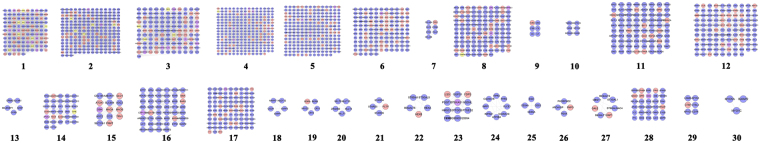
Cluster of compound target-PIH target-human other proteins’ PPI network (Blue circles, pink circles, yellow circles and purple circles stand for other human proteins, compound targets, compound-PIH targets, PIH targets, resp).

Analyze the network by MCODE, thirty-eight clusters are returned (Fig. 7). Deal with these clusters by GO enrichment analysis. Cluster 9 does not return biological processes. Cluster 7, 8, 12, 13, 14, 16, 18, 19, 20, 21, 22, 24, 25, 26, 27, 28, 29, 30 does not return PIH-related biological processes. The details of the clusters are described in Table S7. And after filtering by Bonferroni <0.05, only cluster 1, 2, 3, 4, 15, 17 contained significant biological processes. Take some PIH-related biological processes in Cluster 1 as an example:

Genes in cluster 1 are related to many biological processes. According to PIH’s etiologies, these biological processes can be divided into 4 modules. (1) Endothelial cell activation and injury: (GO:0002576) platelet degranulation, (GO:0030168) platelet activation. (2) Placental or trophoblast cell ischemia: (GO:0045429) Positive regulation of nitric oxide biosynthetic process, (GO:0001525) Angiogenesis, (GO:0051000) Positive regulation of nitric-oxide synthase activity, (GO:0035924) Cellular response to vascular endothelial growth factor stimulus, *et al*. (3) Hypoxia and oxidative stress: (GO:0071456) Cellular response to hypoxia, (GO:0001666) Response to hypoxia, (GO:0034097) Response to cytokine, (GO:0019221) Cytokine-mediated signaling pathway. (4) Maternal-fetal immune tolerance disorders: (GO:0071347) Cellular response to interleukin-1, (GO:0030217) T cell differentiation, (GO:0050852) T cell receptor signaling pathway. The details are described in Table S8.

This network is an extension of the previous, which is established to observe the relationship between predicted targets, PIH targets, and other human proteins. Through this network, we not only find the same biological processes of previous, but also discover some new PIH-related biological processes, such as (GO:0030217) T cell differentiation, (GO:0050852) T cell receptor signaling pathway, *et al*. This is a supplement to the previous one, and it allows us to understand the mechanism of ASC on PIH better. Overall, there are 10 biological processes in the biological process of ASC (Table S2 and S3) overlapping with the biological process of PIH (Table S1). Three of them belongs to “endothelial cell activation and injury” model, including GO:0002576, GO:0030168, GO: 0007596; while seven of them belongs to “placental or trophoblast cell ischemia” model, including GO:0045429, GO:0001525, GO:0051000, GO:0035924, GO:0048010, GO:0045766, GO:0043066. ASC may regulate these biological processes and their genes directly to treat PIH. As for the biological processes of ASC that does not overlap with that of PIH, ASC may indirectly act on them to treat PIH or may not—this, therefore, needs further research to confirm or revise it.

Latest research has shown that some of the compounds in Salvia and Astragalus have a certain effect on the body, which indirectly confirms some of the biological processes we found. For instance, Salvia extracts, including cryptotanshinone and tanshinone IIA are able to remove peroxides (like GO:0001666, GO:0071456), and inhibit expression of adhesion molecules, platelet aggregation (such as GO:0030168, GO:0002576, GO:0007596) and apoptosis (such as GO:0043066)^39^. Tanshinone IIA can also against any vascular disease, such as atherosclerosis and hypertension (like GO:0051000, GO:0050999)^40^, which suggests that it may relieve PIH through anti vascular abnormalities. Kaempferol of Astragalus can inhibit PGD2 and remove various radicals and superoxide radical (like GO:0001666, GO:0071456)^41–43^. In addition, quercetine of Astragalus is an antioxidant, in some pre-eclampsia animal models, adverse outcomes, such as proteinuria and high neonatal death rate, can be reversed by it^44^. These effects may be related to treating PIH.

The core of pregnancy hypertension is characterized by increased uterine spiral arterial vascular resistance in the placenta caused by hypercoagulable state, and a variety of cytokines, inflammatory factors induced vascular endothelial cell apoptosis and vascular remodeling^45^. Inflammatory cytokines play a critical role in platelet adhesion and aggregation as well as placental tissue damage^46^. When these inflammatory cytokines (ROS, O2-, ONOO-, etc.) are activated, they do not only oxidize DNA, proteins and lipids, but also interfere with important vascular-related signaling pathways^47^. NO is an endothelial-derived strong vasodilator and anticoagulin that helps maintain angiotasis, increases uterine blood flow and during pregnancy increases estrogen^48^. Inflammatory factors can induce leukocyte and platelet adhesion to endothelial cells, and the release of cytokines (TNF-α, IL-1, IL-6, INF-γ etc.)^46,49,50^, and antiangiogenic factors (NO). This eventually results in placental endothelial dysfunction.

Our research also found that ASC may have certain effects in regulating VEGF-related biological processes, such as “(GO:0048010) VEGF signaling pathway” and “(GO:0035924) Cellular response to VEGF stimulus”. Angiogenesis imbalance plays an important role in PIH. In the process of angiogenesis, VEGF is considered to be the most potent pro-angiogenic protein^51^. In addition, it can improve renal hemodynamics and reduce proteinuria by significantly inhibiting hypertension to maintain endothelial function^24,26,27^. Endothelial dysfunction and vascular remodeling caused by VEGF deficiency are also one of the core features of PIH.

Currently, as to PIH, perfect treatment has not been discovered. The pharmacologic options include aspirin, antispasmodic drugs (magnesium sulfate) and antioxidants and anti-hypertensive drugs. Herbal medicine is one of many patients’ options. In this study, a number of network-based computational methods and algorithm-based approaches to predict targets, construct networks are combined to explore the molecular mechanism of ASC for PIH. This network pharmacological approach learns many methods from previous TCM network pharmacological research^52–56^, such as pharmacokinetics prediction method^52^, PIH target collection method^53^, cluster analysis^54^ as well as other network analysis methods^55,56^. Thus, compared with previous methods of TCM network pharmacology, this one may combine the advantages of many methodologies that help to make the results more accurate. This method not only supplies a reference for the researcher who is to explore ASC’s therapeutic mechanisms for PIH, but also provides clues to the researcher who explores TCM’s molecular or pharmacological mechanisms.

## Materials and Methods

### Data Preparation

#### Composite Compounds of ASC

To collect the compounds of ASC, we used the TCM Database@Taiwan^57^ (http://tcm.cmu.edu.tw/zh-tw/, updated in March 2014), which is the most comprehensive TCM database in the world; The Traditional Chinese Medicine Systems Pharmacology Database^58^ (TcmSPTM, http://lsp.nwsuaf.edu.cn, updated on May 31, 2014), a unique system pharmacology platform designed for Chinese herbal medicines. Two hundred and two compounds in *Radix Salviae and* eighty-two compounds in *Hedysarum Multijugum Maxim*. were found.

#### Pharmacokinetic Prediction

Due to the disadvantages of biological experiments as time-consuming and high-cost, identification of ADME (absorption, distribution, metabolism and excretion) properties by in silico tools has now become an inevitable paradigm in pharmaceutical research. In this study, three ADME-related models, including the evaluation of oral bioavailability (OB), Caco-2 permeability and drug-likeness (DL), were employed to identify the potential bioactive compounds of ASC.

Oral bioavailability. OB prescreening is used to determine the fraction of the oral dose of bioactive compound which reaches systemic circulation in the TCM remedy. Here, a reliable in silico model OBioavail 1.1^59^ which integrates the metabolism (P450 3A4) and transport (P-glycoprotein) information was employed to calculate the OB values of herbal ingredients.

Caco-2 permeability. The Caco-2 cell monolayers are widely applied as standard permeability-screening assay for prediction of the compound’s intestinal absorption and fraction of the oral dose absorbed in humans^60^. The Caco-2 cell permeation values of all molecules are calculated by in silico model using the VolSurf approach^61^.

Drug-likeness evaluation. Drug-likeness is a qualitative profile used in drug design to evaluate whether a compound is chemically suitable for the drug, and how drug-like a molecule is with respect to parameters affecting its pharmacodynamic and pharmacokinetic profiles which ultimately impact its ADME properties^62^. In order to identify drug-like compounds, we apply a database-dependent model using the Tanimoto coefficient to calculate the DL (see Eq. (1)) of each compound in ASC.

1 $${\rm{f}}(x,y)=\frac{xy}{{|x|}^{2}+{|y|}^{2}-xy}$$

*x* represents the molecular parameters of herbal ingredients, and *y* represents the average molecular properties in DrugBank database (available online: http://www.drugbank.ca).

The OB, Caco-2 permeability and DL of all compounds are described in Table S9.

In this work, the compounds of OB ≥ 30%, Caco-2 > −0. 4 and DL ≥ 0.18 were selected for subsequent research, others are excluded.

According to these indexes, several compounds were included: 1,2,5,6-tetrahydrotanshinone, 3-beta-Hydroxymethyllenetanshiquinone, 3α-hydroxytanshinone IIA, 4-methylenemiltirone, 537-15-5, 87112-49-0, 97399-70-7, 97411-46-6, C09092, Cryptotanshinone, Dan-shexinkum D, Danshenol A, Danshenol B, Danshenspiroketallactone, Dehydrotanshinone IIA, Deoxyneocryptotanshinone, Dihydrotanshinlactone, Dihydrotanshinone I, Epidanshenspiroketallactone, Formyltanshinone, Isocryptotanshinone, Isoimperatorin, Isotanshinone II, Luteolin, Manool, Methylenetanshinquinone, Microstegiol, Miltionone I, Miltionone II, Miltipolone, Miltirone II, Miltirone, MOL007155, MOL007140, MOL007036, MOL007048, MOL007050, MOL007070, Neocryptotanshinone II, Neocryptotanshinone, NSC122421, Poriferast-5-en-3beta-ol, Poriferasterol, Przewaquinone E, Przewaquinone F, Prolithospermic acid, Przewalskin A, Przewalskin B, Przewaquinone B, Przewaquinone C, Salvianolic acid G, Salvilenone I, Salvilenone, Salviolone, Sclareol, Sugiol, Tanshinaldehyde, Tanshindiol B, Tanshinone VI, Tanshinone IIA, α-Amyrin, 1,7-Dihydroxy-3,9-dimethoxy pterocarpene, 3,9-di-O-methylnissolin, 64474-51-7, 64997-52-0, 7-O-methylisomucronulatol, 73340-41-7, Bifendate, Calycosin, Formononetin, Hederagenin, Isodalbergin, Isorhamnetin, Jaranol, Kaempferol, Mairin, Quercetin.

#### Compound Target for ASC

Input all the active compounds into SciFinder (http://scifinder.cas.org), a database of chemical and bibliographic information attached to the Chemical Abstracts Service; and got the molecular structure of each active compound. Draw them in ChemBioDraw and save as “mol2” file format. Import these files into PharmMapper (http://lilab.ecust.edu.cn/pharmmapper/, updated in September 2012), which is a web server for potential drug target identification using pharmacophore mapping approach^63^. Because of the non-standard naming, we use UniProtKB (http://www.uniprot.org/), which the central hub for the collection of functional information on proteins, with accurate, consistent and rich annotation. Input the protein names with the species limited to “Homo sapiens” and we can receive their official symbol. After these operations, proteins information of compound targets and known targets was obtained. The details are described in Table S2 (see Supplementary Materials).

#### PIH Targets

We collected different genes associated with PIH from tow resources. (1) Genecards (http://www.genecards.org), it is a database about genes, their products and biomedical applications, which is maintained by Israel’s Weizmann Institute of science. (2) OMIM database (http://omim.org/), which catalogues all known diseases with a genetic component and when possible links them to the relevant genes in the human genome and provides references for further research and tools for genomic analysis of a catalogued gene^64^.

We searched these databases with keywords “Pregnancy-Induced Hypertension”, “Gestational Hypertension”, “Pregnancy Transient Hypertension” and got 131 genes totally. The details are described in Table S10.

#### Other Human Protein and Protein-Protein Interaction Data

The data of other human protein and protein-protein interaction (PPI) came from both InAct^65^ and String^66^, with the species limited to “Homo sapiens” and a confidence score >0.4.

String (http://string-db.org/, ver. 10) is a database of known and forecasted protein-protein interactions and InAct(http://www.ebi.ac.uk/intact/, ver. 4.2.4) provides an open source database and analysis tools for molecular interaction data.

### Network Construction

#### Network Construction Method

All the networks could be created via utilizing the network visualization software Cytoscape^67^ (http://cytoscape.org/, ver. 3.4.0). It is the software that applies to visualizing biological pathways, intermolecular interaction networks and many more. Furthermore, it supplies a basic set of features for data integration, analysis, and visualization for complicated network analysis.

Input the targets and the data of PPI into Cytoscape to construct different networks based on this research. Network construction was performed as follows: (1) PIH network; (2) Compound-compound target network of ASC; (3) ASC-PIH network; (4) Compound target-PIH target-other human proteins’ PPI network.

#### Cluster

The densely connected regions in large protein-protein interaction networks that may represent molecular complexes is defined as topological modules or clusters^68,69^, which has pure network property. Aggregation of nodes of similar or related function in the same network is called functional modules. A group of network components that together disrupt cellular function and then results in a particular disease phenotype are disease module. Due to that the topology module, functional module and disease module have the same meaning in the network, the functional module is equal to topology module and the disease can be regarded as the disturbance and destruction of functional model^68^. The clusters of each network were obtained by analyzing the corresponding networks by MCODE, a plug-in of Cytoscape^69^.

### Gene Ontology enrichment analysis

The Database for Annotation, Visualization and Integrated Discovery^70^ (DAVID, https://david-d.ncifcrf.gov, ver. 6.8) was applied for Gene Ontology (GO) enrichment analysis. The biological process that Bonferroni <0.05 were thought to be a significant biological process.

## Conclusions

ASC may directly regulate several biological processes and their genes in “endothelial cell activation and injury” and “placental or trophoblast cell ischemia” models to treat PIH. And it may indirectly act on the rest of the biological process to treat PIH or may not.

## Electronic supplementary material

Table S1

Table S2

Table S3

Table S4

Table S5

Table S6

Table S7

Table S8

Table S9

Table S10
